# Gene Expression Profiles of the NCI-60 Human Tumor Cell Lines Define Molecular Interaction Networks Governing Cell Migration Processes

**DOI:** 10.1371/journal.pone.0035716

**Published:** 2012-05-03

**Authors:** Kurt W. Kohn, Barry R. Zeeberg, William C. Reinhold, Margot Sunshine, Augustin Luna, Yves Pommier

**Affiliations:** Laboratory of Molecular Pharmacology, Center for Cancer Research, National Cancer Institute, NIH, Bethesda, Maryland, United States of America; University of Nebraska Medical Center, United States of America

## Abstract

Although there is extensive information on gene expression and molecular interactions in various cell types, integrating those data in a functionally coherent manner remains challenging. This study explores the premise that genes whose expression at the mRNA level is correlated over diverse cell lines are likely to function together in a network of molecular interactions. We previously derived expression-correlated gene clusters from the database of the NCI-60 human tumor cell lines and associated each cluster with function categories of the Gene Ontology (GO) database. From a cluster rich in genes associated with GO categories related to cell migration, we extracted 15 genes that were highly cross-correlated; prominent among them were RRAS, AXL, ADAM9, FN14, and integrin-beta1. We then used those 15 genes as bait to identify other correlated genes in the NCI-60 database. A survey of current literature disclosed, not only that many of the expression-correlated genes engaged in molecular interactions related to migration, invasion, and metastasis, but that highly cross-correlated subsets of those genes engaged in specific cell migration processes. We assembled this information in molecular interaction maps (MIMs) that depict networks governing 3 cell migration processes: degradation of extracellular matrix, production of transient focal complexes at the leading edge of the cell, and retraction of the rear part of the cell. Also depicted are interactions controlling the release and effects of calcium ions, which may regulate migration in a spaciotemporal manner in the cell. The MIMs and associated text comprise a detailed and integrated summary of what is currently known or surmised about the role of the expression cross-correlated genes in molecular networks governing those processes.

## Introduction

Although a great deal of information has accumulated on gene expression and molecular interactions in various cell types, relating those data to cell functions remains challenging. Here we ask whether that relationship can be fruitfully probed on the basis of gene expression profiles of a set of diverse human tumor cell lines. Malignant cells often retain histological characteristics resembling the tissue of origin, and tumor cell lines derived from the same tissue of origin often retain similar gene expression patterns [1], [2], [3]. Therefore groups of genes that are expressed specifically in tumor cell lines from one or more tissues of origin may reflect some aspect of the cells' “life-styles”. Cell lines having epithelial versus mesenchymal characteristics, for example, have gene expression patterns that tend to correspond to those respective tissue types (reference [4] and K. W. Kohn and B. R. Zeeberg unpublished data). Mutations and genome scrambling in malignant tumors, however, can cause gene expression patterns to diverge substantially among different cell lines of a given tissue type.

The NCI-60 are a set of 60 human tumor cell lines derived from various tissues of origin. Expression of approximately 16,000 genes in each of those cell lines has been assayed and subjected to bioinformatic analyses [3], [5], [6], [7], [8]. We recently developed a procedure that generated gene clusters based on NCI-60 gene expression profiles and that associated the gene clusters with sets of function categories defined by the Gene Ontology (GO) database [9]. We focus here on one of those clusters (cluster 52/160), which was rich in genes associated with GO categories related to cell migration.

The ability to migrate and invade normal tissues inappropriately is one of the features that tumor cells must acquire to become fully malignant [10]. The mobility of malignant tumor cells depends on complex molecular interactions that regulate the structure, function, and interactions of cytoskeleton and extracellular matrix [11]. Here we describe an expression-correlated set of genes that function in molecular interaction networks promoting cell migration through extracellular matrix degradation and calcium signaling. We depict the networks using our notation for molecular interaction maps [12], [13]. The results organize the available current information about those processes and suggest new viewpoints, as well as new functional relationships for investigation. To our knowledge, this is the most detailed description and mapping so far reported of molecular interaction networks linked to gene expression data relevant to mammalian cell migration.

## Methods

### CellMiner, cluster analysis, and derivations

The mRNA expression data for the NCI-60 human tumor cell lines were retrieved from CellMiner relational database version 1.0 [14], which will become freely available (contact William Reinhold: wcr@mail.nih.gov). The database contains transcript expression values for several assays of the 60 cell lines, which are normalized to generate expression profiles called “z-scores.” Gene transcript expression values were used as described by Zeeberg et al. [9].

**Figure 1:**
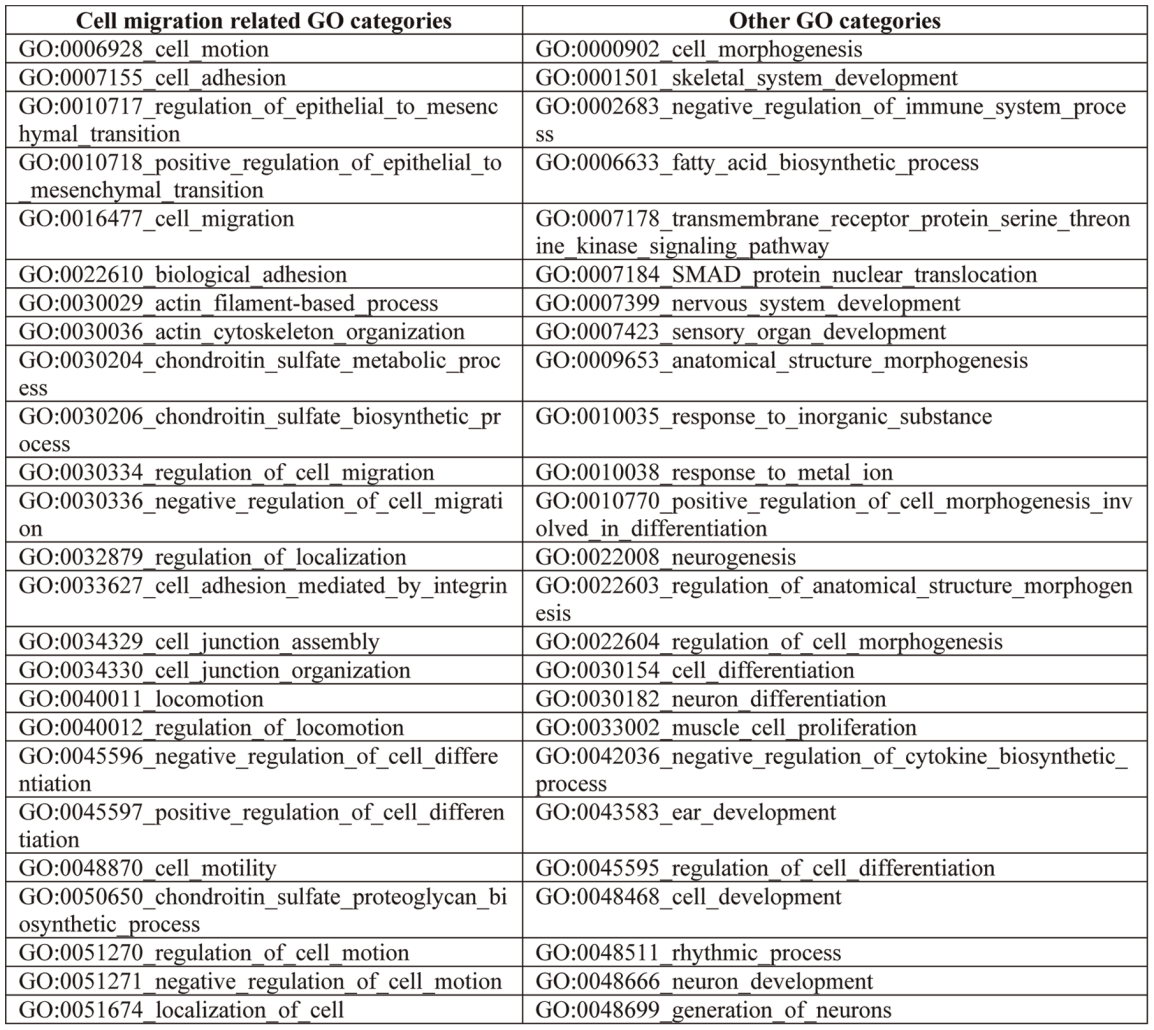
Gene Ontology (GO) categories associated with the 83 genes in cluster **52/160 (see** ***Methods*** **).**

The genes studied in the current work were based on an NCI-60 gene expression cluster,designated cluster 52/160, that was identified by Zeeberg et al. [9]. In brief, gene clusters based on expression in the NCI-60 cell lines were derived at 4 levels of resolution (20, 40, 80, and 160 clusters) such that a cluster in the 160 level, for example, was a “child” (and therefore a subset) of some cluster in the 80 level. The 300 gene lists corresponding to each cluster were then submitted to the High-Throughput GoMiner (HTGM) program [15] to determine the corresponding Gene Ontology (GO) [16], [17] functional categories. The current work investigates one of the highest resolution clusters (designated cluster 52/160 – cluster 52 of the 160 level), a cluster associated with GO categories, many of which are related to cell adhesion and migration (Figure 1).

### High cross-correlation set (HCCS) genes

**Figure 2:**
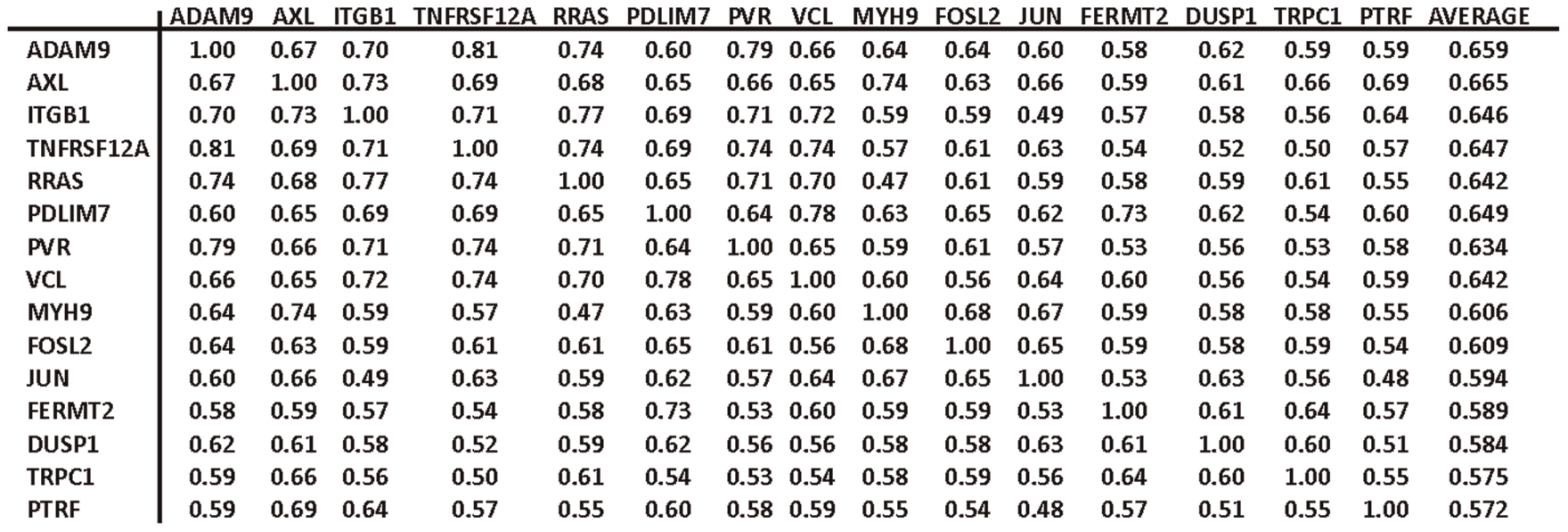
High cross-correlation set of 15 genes (HCCS15) derived from cluster 52/160. The genes marked with an asterisk (*) are discussed in the current report and are included in the molecular interaction maps (Figures 11 and 12). The other genes in the table function in other aspects of cell migration that are not covered in the present report. The numbers in the table are expression profile correlations for expression of gene pairs in the NCI-60 human tumor cell lines (expression profile correlation >0.50 corresponds approximately to P<0.001; expression profile correlations >0.60 are almost never due to chance alone).

From the 83 genes in cluster 52/160, we found 15 genes (“HCCS15”, Figure 2) that were highly cross-correlated with each other. HCCS15 is the set of all cluster 52/160 genes such that the expression profile of each gene was correlated with all the other genes in the set, the required pair-wise correlation between expression profiles being >0.50 (corresponding approximately to P<0.001) (a rare value between 0.47 and 0.50 was permitted). Those 15 genes were then used as seeds to find other genes with similar expression profiles across the NCI-60 cell lines (NCI's CellMiner software, under development). The resulting set of 66 high cross-correlated genes (“HCCS66”, Figure S1) contains 51 additional genes selecte on the basis of correlation with all of the original HCCS15 genes, such that all expression profile correlations were greater than 0.40 and that at least 11 expression profile correlations were >0.50, and further that average expression profile correlation over the entire set of 66 other genes in HCCS66 was >0.50. (Many of the additional 51 genes met correlation criteria that might have included them in cluster 52/160, but the clustering algorithm, which assigned each gene to only one cluster, assigned them to other clusters with which they were at least as well correlated [9]).

### Literature survey

PubMed literature surveys for relevant genes focused on the most recent publications of new findings and reviews. In order to keep the reference list as short and relevant as possible, recent articles were often cited that contained references to the original work. In those rare instances of substantial divergence of reports, we accepted the inferences when recent reports indicated a consensus of opinion. The PubMed searches included alternative names of the genes or gene products. We generally use the HUGO Gene Nomenclature Committee (HGNC) names for genes [18], although we sometimes used common names for the expressed proteins. It was often useful to search for papers dealing with a combination of two genes or gene products to look for data indicating interactions between the two. We used direct literature surveys rather than indirect literature search software in order to obtain the most detailed and up-to-date information. Sometimes gene pairs having highly correlated expression profiles were not mentioned together in any publication, suggesting that future studies may disclose new pathways involving those gene pairs.

### Molecular interaction maps (MIM)

Molecular interaction maps (MIMs) were created using a Pathvisio-based tool recently developed in our Laboratory to assist drawing MIM-type diagrams [19]. The symbols and rules of the MIM notation are described in [13] and at http://discover.nci.nih.gov/mim/. The molecular interactions in the MIM diagrams are numbered for reference in the text.

Our MIMs are intended to show the interactions that would occur when the relevant molecular species are present at the same time and place. Expression and localization must therefore be taken into account in applying MIMs to particular biological situations.

## Results

In order to select a group of genes that are likely to engage in molecular interactions leading to a particular function, we began with the 83 genes that constitute cluster 52/160 of Zeeberg et al [9], derived on the basis of similarity of gene expression profile across the NCI-60 cell lines. This set of genes mapped to many GO categories related to cell migration, as well as to categories related to other functions (Figure 1). We used the genes in cluster 52/160 as a vehicle for assembly of expression-correlated genes that co-function in molecular interaction networks describing particular aspects of cell migration control.

**Figure 3:**
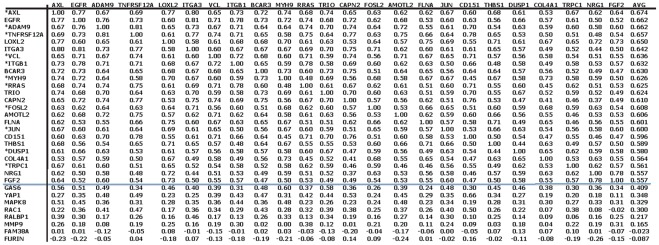
Expression profile cross-correlations of the 32 genes that participate in the interaction networks shown as molecular interaction maps in **Figures 11** and **12**. The right-most column lists the averages for each row. Above the horizontal line are 24 genes (HCCS24) that had expression profile cross-correlation averages greater than 0.50; these 24 genes are shown in red in Figures 11 and 12. Genes marked with an asterisk (*) were also in the HCCS15 gene set (Figure 1). Below the lines are genes that, although they participate in the networks shown in Figures 11 and 12, had expression profile correlations too low to be included in HCCS66 or HCCS24; these genes are shown in black in Figures 11 and 12. (Individual expression profile correlations >0.30 were considered significant.)

**Figure 4 pone-0035716-g004:**
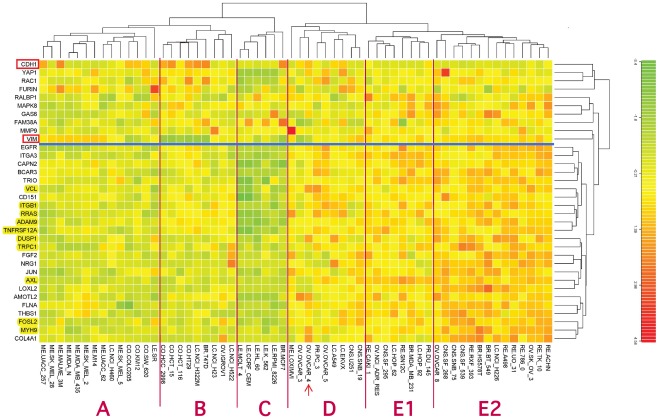
Clustered image map (CIM) of the relative expression of the 32 genes in **Figure 3** in each of the NCI-60 cell lines. In addition, the CIM includes CDH1 (E-cadherin, a marker genes for epithelial character) and VIM (vimentin, a marker genes for mesenchymal character). The CIM image (Euclidean norm, complete linkage) was computed using custom in-house R language code. The genes below the blue line are the 24 genes in high cross-correlation set HCCS24 (above the blue line in Figure 3 and marked red in Figures 11 and 12 ). Genes that were also in HCCS15 (Figure 2) are highlighted in yellow. The vertical red lines help to discern which cell lines of a given tissue type cluster together, which deviate, and which fall into a cluster dominated by a different tissue type. The arrow points to ovarian carcinoma cell line OVCAR4, which was unusual in showing high expression of both CDH1 and VCL.

**Figure 5:**
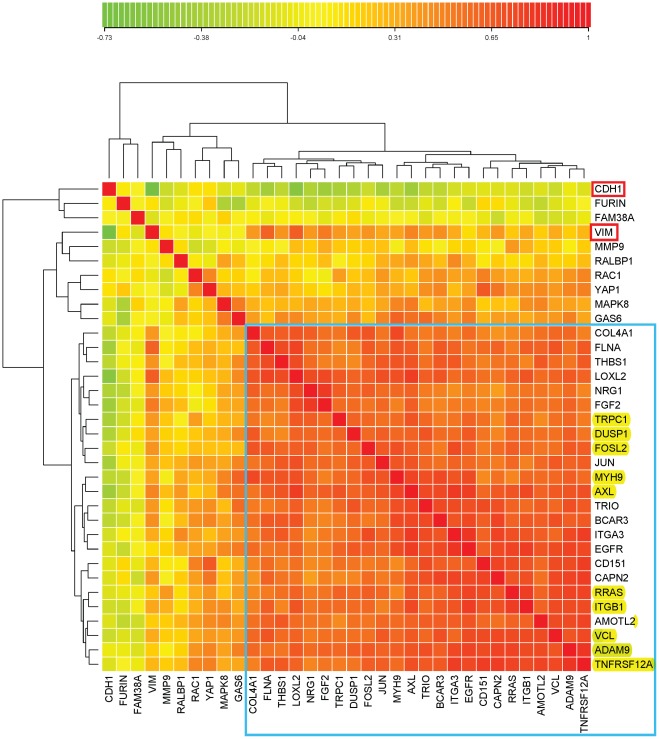
Correlation CIM of the same genes as in Figure 4. The genes in the blue box are the 24 high cross-correlation HCCS24 genes; these genes correlated significantly with VIM and not with CDH1, showing that they correlate with mesenchymal character. Note however that some genes correlated better than others with VIM: genes such as RAC1 and GAS6, as well as VIM, which failed to meet the strict criteria for inclusion with the HCCS24 genes, nevertheless are seen to be significantly correlated with them. Interestingly, some of the genes that were imperfectly correlated with the HCCS24 genes (i.e., GAS6, MAPK8, YAP1, and RAC1) tend to correlate inversely with VIM; the cell lines that contribute to this effect can be seen in Figure 4 (they include 4 colon lines that express CDH1 but not VIM). The low correlation of FURIN with the HCCS24 genes is attributable to its functioning in stromal rather than tumor cells (Figure 12); the low correlation of FAM38A however remains unexplained.

From cluster 52/160, we selected 15 genes (HCCS15) whose expression profiles across the NCI-60 cell lines were highly cross-correlated with each other (Figure 2). The criterion for inclusion in HCCS15 was that each gene have expression profile correlation of at least 0.50 with respect to every other gene in the set. To this set of 15 genes, an additional 51 were added on the basis of highly correlated expression in the NCI-60 cell lines, as described under *Methods*. The data for the complete set of 66 genes (HCCS66) are presented as a clustered image map (CIM) (Figure S1). For most of these 66 genes, survey of current literature disclosed evidence of molecular interactions related to some aspect of cell migration or invasion, including (1) integrin and cytoskeleton-targeted actions of RRAS and calcium; (2) degradation and remodeling of extracellular matrix; (3) retraction of the rear of a migrating cell; (4) connections and remodelling between integrins and actin cytoskeleton; (5) role of endocytosis of cell surface receptors; and (6) role of proteoglycans in extracellular matrix. The current report focuses on the first three of these. Among the 66 genes in the cross-correlated set HCCS66, we found 24 that had functions related to the 3 aspects of cell migration covered by this report. These 24 genes (HCCS24) are listed above the blue line in Figure 3, together with their pair-wise expression profile correlations, and will appear in red in the molecular interaction maps. Data for an additional 8 interacting genes, derived from the literature survey, are listed below the horizontal blue line in Figure 3; the cross-correlations for these 8 genes were not sufficiently high to be included in HCCS66 or HCCS24. A clustered image map (CIM) of the relative expression of the 32 genes in each of the NCI-60 cell lines is shown in Figure 4, and a gene correlation CIM is shown in Figure 5.

### RRAS and calcium-dependent interactions involving integrins and actin cytoskeleton

**Figure 6:**
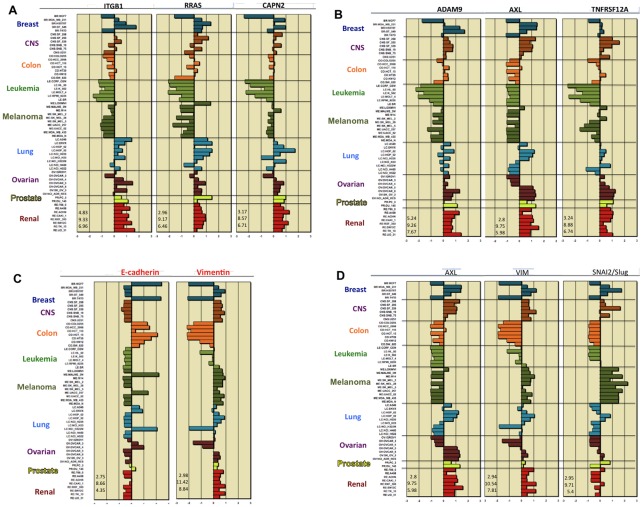
Relative gene expressions for the NCI-60 human tumor cell lines. Bars to the right show increased expression, bars to the left show decreased expression relative to the expression mean. Each panel shows the lowest, highest and mean expression levels (numbers at the lower left from top to bottom). Expression values are normalized as z-scores (see Methods). The horizontal axes are marked in standard deviations from the mean. (A) Expression profiles for RRAS, ITGB1, and CAPN2. (B) Expression profiles for ADAM9, AXL, and TNFRSF12A. (C) Epithelial and mesenchymal gene expression profiles, indicated by CDH1 (E-cadherin) and VIM (vimentin) respectively, showing their inverse relationships. (D) Comparison of expression profile of migration-related genes, represented by AXL, with that of mesenchymal-related genes, represented by VIM and SNAI2 (Slug).

**Figure 7:**
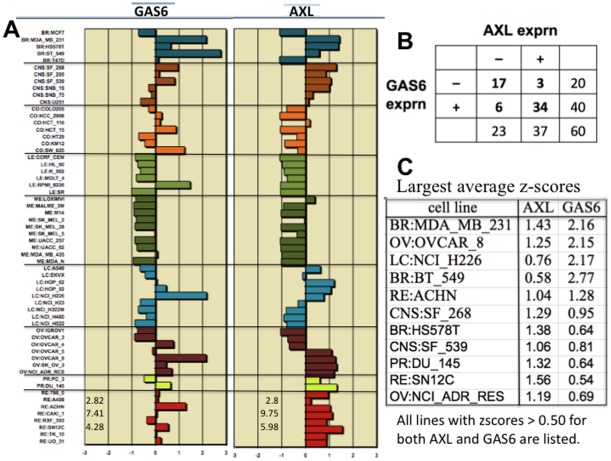
Expression of AXL and its ligand GAS6. (A) Expression level (z-score) of each cell line. (B) Number of cell lines that express AXL, GAS6, both, or neither. (C) Cell lines that strongly express both AXL and GAS6, suggestive of a possible autocrine mechanism.

Having assembled the subset of 15 high cross-correlated genes (HCCS15) from the 83 genes of cluster 52/160, we noted that the expression profile correlation (0.78) between **RRAS** and **ITGB1** (beta1-integrin) was among the highest we had seen (Figure 2). Although RRAS and ITGB1 did not at first seem functionally related, a recent report showed how they may function together in a well-defined network [20]. We found that report by a literature search only after having observed the unexpected high expression correlation. That encouraged us to pursue the premise that mRNA expression correlations in the NCI-60 cell lines can predict functional relationships. The expression profiles of several key genes are shown in figures 6 and 7.

ITGB1 and RRAS were highly expressed in all of the renal and most of the lung cancer cell lines, and both genes were expressed at relatively low or undetectable levels in all of the leukemia and most of the melanoma cell lines (Figure 6A). The NCI-60 data show that every cell line that expressed ITGB1 also expressed RRAS. When ITGB1 is expressed, therefore, an RRAS-dependent process to regulate its function may commonly be in place. Of 27 lines that showed substantial RRAS expression, T47D was the only one that failed to express ITGB1 (Figure 6A). Therefore, regulation of ITGB1 could be an important RRAS function.

**Figure 8:**
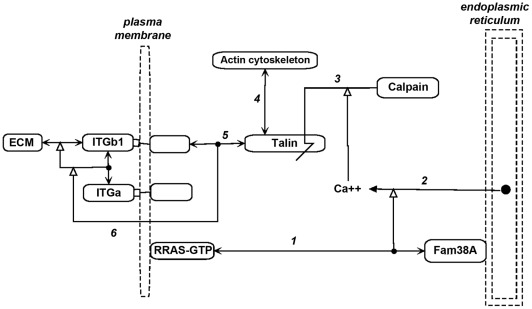
Molecular interaction map (MIM) of the role of RRAS and calcium in the regulation of linkage between integrins and actin cytoskeleton, as proposed by McHugh et al [**20**] **.** ECM, extracellular matrix.

**Figure 9:**
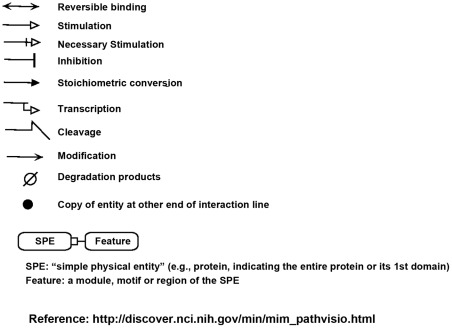
Essential molecular interaction map (MIM) symbols. For detailed descriptions of the MIM notation, see [13] or http://discover.nci.nih.gov/mim/mim_pathvisio.html

The mechanism proposed by McHugh et al [20] is portrayed as a molecular interaction map (MIM) in Figure 8. (MIM symbols are summarized in Figure 9.) In their proposal, Fam38A recruits activated RRAS to the endoplasmic reticulum (ER) (*interaction 1*) and causes Ca (2^+^) release from the ER (*interaction 2*). The increased cytosolic Ca (2^+^) then activates Calpain to cleave Talin (*interaction 3*), thereby disrupting the linkage between integrins and the actin cytoskeleton (*interactions 4 and 5*). Consequently, the stable linkage between integrin and extracellular matrix (ECM), which is dependent on inside-out-signaling [11] (*interaction* 6), is lost.

The model was supported by the finding that tight linkage between integrin and extracellular matrix (ECM) was blocked by inhibiting Fam38A, RRAS, or Talin [20]. Also consistent with the model was the finding that endocytosis of ITGB1, after it has been freed from ECM attachment, was RRAS-dependent [21], and that epithelial cell migration requires RRAS [22]. This pathway may be regulated by activation/inactivation of RRAS, the mechanism of which remains to be elucidated.

**Figure 10:**
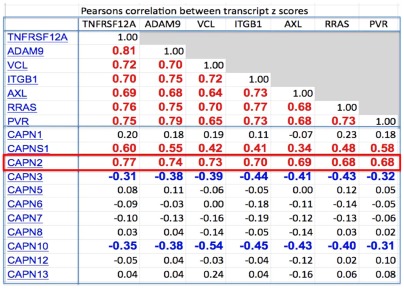
Expression profile correlations of calpain family genes and key genes the HCCS15 high cross-correlation subset. Expression data for CAPN4 and CAPN11 were not available. CAPNS1 is known to function together with CAPN2.

We then looked to see whether the NCI-60 expression profiles implicated Fam38A, or any of the calpains or talins. Among those genes, **CAPN2** (calpain-2) gene expression stood out in being strongly correlated with the HCCS15 genes (Figures 3 and 6A). CAPN2 was not in the original cluster 52/160 only because the clustering algorithm assigned it to another gene cluster where its correlations were at least as good [9]. Of the other calpains, only CAPNS1, which co-functions with CAPN2, showed high correlations (Figure 10). Neither Fam38A nor any of the talins showed high correlation. We infer therefore that CAPN2 is probably the calpain that operates in the RRAS-ITGB1 pathway proposed by McHugh et al [20]. CAPN2 is activated at relatively high Ca (2^+^) concentrations (0.4–0.8 mM) and is thought to cleave focal adhesion components to facilitate rear retraction [23].

**Figure 11:**
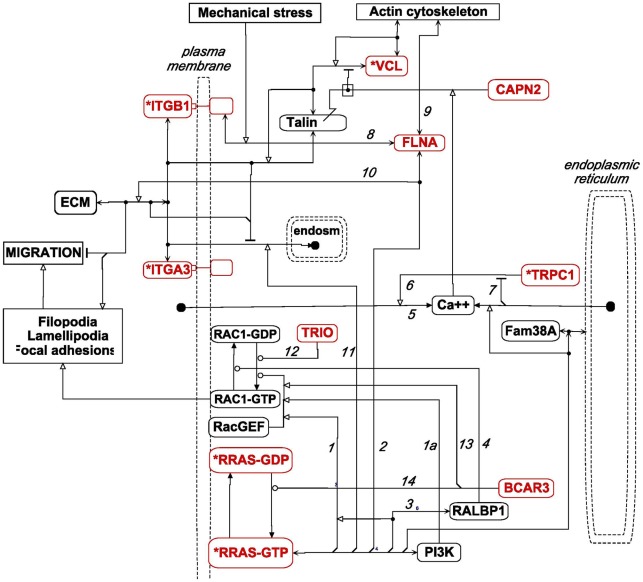
MIM of the role of RRAS and calcium in the regulation of linkage between integrins and actin cytoskeleton, including participation of relevant genes from the HCCS66 gene set. HCCS66 genes are colored red; those from the original HCCS15 set are also marked with an asterisk. The interactions of Fam38A and CAPN2 are described in Figure 8 and related text. ER, endoplasmic reticulum. (MIM symbol definitions are summarized in Figure 9.)

We then extended the McHugh et al model by adding relevant genes from the high cross-correlation gene set HCCS66, as shown in the MIM in Figure 11; the interactions and functions for each gene are summarized below. Note that all of the HCCS24 genes in Figure 11 (red), with the exception of TRPC1, are included in the same gene cluster in the expression CIM (Figure 5, blue box).

**RRAS** participates in multiple functions, several of which are relevant to the regulation of integrin-cytoskeleton linkage and which are depicted in Figure 11. RRAS regulates cell migration and adhesion, although the effects may be stimulatory or inhibitory, apparently depending on cell type or conditions [24].

RRAS can promote cell migration by activating RAC1 through stimulation of a guanine nucleotide exchange factor (GEF) (Figure 11, *interaction 1*). This may be mediated through RRAS binding and activating phosphatidylinositol-3–kinase (PI3K), since a PI3K inhibitor (LY294002) inhibited RRAS-induced RAC1 activation [25]. RRAS binds FLNA (filamin A) (*interaction 2*), and this combination was required for migration of melanoma cells [26] (see section below on FLNA).

Cell migration and spreading due to activation of RAC1 by RRAS is dependent on RALBP1/RLIP76 (*interaction 3*) [27]. However, RALBP1 is also a GTPase activating protein (GAP) that inactivates RAC1 by converting the GTP to the GDP form (*interaction 4*) [28]. The latter effect may seem contradictory, but may reflect a cyclic process of cell attachment/detachment from ECM.

***TRPC1***, a HCCS15 gene (Figure 2), is a component of calcium channels in the plasma membrane that are activated by depletion of calcium stores in the endoplasmic reticulum (ER) [29]–[30] (*interactions 5–7*). ER calcium concentration is sensed by STIM1, which activates TRPC1 when ER calcium concentration is low [31]). TRPC1 forms heterodimers with other TRPC's to constitute store-dependent cation channels. However none of the other TRPC genes were well correlated with HCCS genes. The high expression correlation between TRPC1 and RRAS within the HCCS (zscore = 0.62, Figure 2) suggests that these 2 genes function in concert to regulate calcium concentration in migrating cells, *i.e*., RRAS permitting calcium release from endoplasmic reticulum and TRPC1 allowing calcium entry into the cell if endoplasmic reticulum calcium is depleted. The combination of transient calcium release from endoplasmic reticulum and calcium entry through plasma membrane pores might produce calcium flickers (duration of a few seconds or less) near the leading edge of a migrating cell [23]. In addition, the migrating cell exhibits a more persistent and spacially extended calcium gradient increasing from front to rear. The possible role of RRAS-FAM38A-TRPC1 in these calcium patterns remains to be elucidated.

**FLNA** (filamin A), was not a member of the HCCS15 genes, but correlated strongly with those genes, and was in the extended set HCCS24 of highly cross-correlated genes represented in the molecular interactions map in Figure 11. FLNA promotes cell motility and migration, binds beta-integrin tails, and associates and co-localizes with RRAS [26]. FLNA also binds non-muscle actin, thus functioning as scaffold for F-actin, integrins, and RRAS (Figure 11, *interactions 2, 8, and 9*). FLNA and RRAS are required for migration of at least some cell types [26]. The RRAS-FLNA combination can increase the fibronectin-binding affinity of integrins and enhance fibronectin matrix assembly [26] (*interaction 10*). Additionally, endocytosis of ITGB1 from ruffles was recently found to be RRAS-dependent [21] (*interaction 11*).

Where FLNA is abundant RRAS thus may enhance integrin attachment to ECM, whereas in regions of the cell where FLNA is lacking RRAS may stimulate calcium release and abrogate those attachments. This may implement the dynamic attachment to and release from ECM at local regions of the cell surface, which is required for cell mobility (see *Discussion*).

**TRIO**, a member of the HCCS24 gene set (Figures 3, genes above the blue line), is a guanine nucleotide exchange factor (GEF) that activates RAC1 (Figure 11, *interaction 12*) and stimulates the invasive behavior of some glioblastoma and breast cancers; inhibitors of this action of TRIO have been developed [32]–[33]. RAC1, as well as RRAS, may also be activated indirectly by way of HCCS24 gene **BCAR3** (*interactions 13 and 14*) [34].

**VCL** (vinculin) is one of the 15 genes in the original high cross-correlation set HCCS15 (Figure 2). It is a key component of the chain connecting integrins with F-action, most prominently connecting to integrin via talin (Figure 11). Although a great deal of information has been reported about the structure and function of VCL, much remains to be learned about its complex interactions in various locations and under various circumstances [35].

### Modulation of interactions between extracellular matrix and the surface of migrating cells

**Figure 12:**
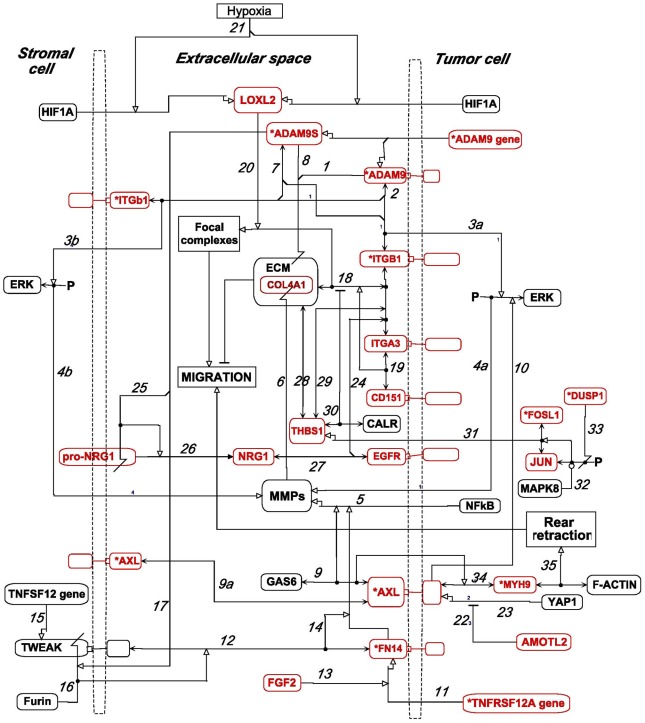
MIM of the role of ADAM9, AXL, ITGB1, TNFRSF12A/FN14 and functionally related genes/proteins in extracellular matrix (ECM) degradation or remodeling. HCCS66 genes are colored red; those that were also in the original HCCS15 set are marked with an asterisk. (MIM symbol definitions are summarized in Figure 9.)

In order to migrate, cells must sequentially connect and disconnect cell surface regions from extracellular matrix (ECM) [36]. Release from ECM may occur through dissolving the connections between binding domains or through degrading or sequestering components of the ECM or cell surface proteins. In some tumor cells, ECM degradation or remodeling is required for the mobility to invade and metastasize [37]. Here we describe how the 4 most highly cross-correlated HCCS15 genes (ADAM9, AXL, ITGB1, and TNFRSF12A/FN14) (Figure 2), as well as some related genes, may function coherently to modulate the binding between ECM and the cell surface (Figure 12).

**ADAM9** encodes an integral membrane protein, located at the basolateral surface of epithelial cells, that promotes degradation of the basement membrane, thereby facilitating cell migration and invasion [38]. It is a metalloproteinase with domains mediating cell-cell and cell-matrix interactions. In addition, it is a major sheddase that releases integrins, as well as EGFR ligand NRG1b, from its membrane-bound precursor into the extracellular medium [39]–[40]. The chromosomal region of ADAM9 (8p11–12) is amplified in some breast cancer tumors and cell lines. Over-expression of secreted short isoform ADAM9S enhances cell migration, whereas expression of full length ADAM9 tends to reduce cell migration [38].

ADAM9 is widely expressed in mammalian tissues, including stem cells [41], is found in all components of the nephron, and interacts with beta-1 integrins at the basolateral surfaces of cells [39]. It is highly expressed in a large fraction of renal cell cancers, as well as other solid tumors, and is associated with metastases and unfavorable prognosis [42] [41]. In accord with those reports, ADAM9 was strongly expressed in 7 of the 8 NCI-60 renal cancer cell lines (Figure 6B).

ADAM9, or secreted isoform ADAM9S, can cleave ECM proteins directly (Figure 12, *interactions 1* and *8*), and can bind ITGB1 (integrin beta-1) on either the same or on an adjacent cell (*interactions 2* and *7*). Additionally, they can induce the expression of matrix metalloproteinases, such as MMP9, which is secreted into the ECM [43]. MMP9 expression requires the Ras-Raf-Erk pathway, which may be activated by the ADAM9-integrin combination, either in the same cell (*interactions 3a* and *4a*) or in an adjacent cell (*interactions 3b* and *4b*). Expression may be further enhanced by transcription factors such as NFkB [37] (*interaction 5*). MMP9 (or other MMPs) then degrade collagen and other ECM proteins, including HCCS66 gene product COL4A1 (*interaction 6*). Both the direct and MMP-mediated protease activities may facilitate cell migration [43].

ADAM9S induces cells to become highly invasive in Matrigel assays, and the protease activity of ADAM9S is required for this effect [44]. ADAM9S secreted by tumor cells may bind integrins on neighboring stromal cells, which rather than the tumor cells themselves may be responsible for most of the protease-induced cleavage of ECM proteins [44]. Up-regulation of ADAM9S may favor separation of adjacent cells and remodeling of ECM. That this may be a key role of ADAM9 in regulating cell migration is consistent with ADAM9 being near the top of the HCCS15 high cross-correlation list (Figure 2).

**AXL** encodes a receptor tyrosine kinase that can be activated by its secreted ligand, GAS6 [45] (Figure 12, *interaction 9*). AXL can bind a variety of intracellular signaling molecules, including PI3K and Grb2. More important however may be its ability to activate the Ras-Raf-Erk pathway leading to induction and secretion of MMP9 (*interaction 10*). This induction is coordinated with NFkB, which is GAS6-independent. MMP9-induction by AXL enhances the invasive capability of tumor cells [37] [46]. Thus, suppression of AXL (by means of shAXL) suppressed the level of MMP9 mRNA [47].

ADAM9 and AXL thus may both input to the Ras-Raf-Erk pathway leading to induction and secretion of MMPs such as MMP9. At least in the case of ADAM9, this may be induced by an inter-cellular interaction that stimulates the pathway in an adjacent stromal cell (“by-stander effect”); stromal fibroblasts are often intimately associated with cancer cells and can form tight junctions and paracrine interactions with them [48], [49].

AXL is often expressed together with GAS6 in a variety of human cancers, often being highly expressed in advanced stage human breast and ovarian cancer [50]. High AXL expression in breast cancer is associated with reduced survival [51], and suppression of AXL prevented the initiation of ovarian metastases in mice [47]; AXL gene expression was moderate to high in 4 of the 7 NCI-60 ovarian cancer lines (Figure 6B).

The stimulation of cell invasiveness by AXL is at least in part mediated via matrix metalloproteinases; AXL-induced cell migration required MMP9, and suppression of AXL (by means of shAXL) suppressed the level of MMP9 mRNA [47].

Although GAS6 did not correlate well in overall gene expression with the HCCS genes, GAS6 and AXL nevertheless were strongly expressed together in several of the NCI-60 cell lines (Figure 7A). Most cell lines expressed both AXL and GAS6 at significant levels or expressed neither gene significantly (Figure 7B). An autocrine mechanism, as previously noted in metastatic cancers [51], may therefore be operating particularly in those cell lines that expressed both genes at high levels (Figure 7C).

Activation of overexpressed AXL can cause cell aggregation, which suggests that AXL may engage in homophilic interactions between neighboring cells (Figure 11, *interaction 9a*). The cell adhesion effect of AXL appears to be ligand-independent and does not require the intracellular domain; this binding may contribute to the ability of cells to metastasize [52].

***TNFRSF12A*** encodes **FN14** (Figure 12, *interaction 11*), a type I membrane protein. It is a receptor for TWEAK (*interaction 12*), a type II membrane protein located at cell-cell junctions [53]. **FGF2**, a HCCS24 gene (Figure 3), enhances the expression of TNFRSF12A [54] (*interaction 13*). It also mediates TWEAK-induced NFkB activity, which enhances the expression of MMP9 [53], [55] (*interaction 14*).

TNFSF12, the gene that codes for TWEAK (interaction 15), is induced in injured tissues and in stroma associated with tumors; increased levels of TWEAK have been found in several types of solid tumors [53]. A secreted form of TWEAK may be generated by proteolyic cleavage, perhaps by Furin convetase (*interaction 16*). Furin is over-expressed in colon, head and neck, and ovarian cancers, and over-expression in ovarian cancer correlated with decreased survival [56]. There was no significant correlation between Furin or TNFSF12 and cell migration-related NCI-60 genes, perhaps because Furin and TNFSF12 act in adjacent stromal rather than in tumor cells.

The interaction of TWEAK with FN14 in cells of the central nervous system increases the permeability of the blood-brain barrier, probably due to degradation of extracellular matrix components by MMP9 [57]. The TWEAK-FN14-induced activation of the NFkB pathway mediates hypoxia-induced neuronal cell death and PARP accumulation in vitro and in vivo [58].

The strongly correlated expression profiles of ADAM9 and TNFRSF12A (Figures 2 and 6B) suggests that ADAM9 may directly or indirectly cleave TWEAK on adjacent cells and release the truncated product from the membrane, making it available for binding to its receptor, TNFRSF12A/FN14. This conjecture is included in the molecular interaction model shown in Figure 12 (*interaction 17*).

Thus the coherent functions of ADAM9, AXL, and TNFRSF12A are remarkably consistent with their highly cross-correlated gene expressions in the NCI-60 cell lines (expression profile correlations 0.67–0.81, Figure 2). The relevant focus of action of these genes/gene products is degradation of extracellular matrix via metalloproteinases, and, interestingly, these 3 genes share the ability to engage in bindings between adjacent cells. To our knowledge, the functional relationship among these genes has not previously been reported.

The cell lines exhibiting the strongest co-expression of ADAM9, TNFRSF12A, and AXL were 2 ovarian cancer lines (OVCAR_8 and SK_OV_3), lung cancer line HOP_92, and several renal cancer lines, suggesting that these cell lines may be particularly active in degrading extracellular matrix. These cell lines may be suitable for investigation of the function relationships of these genes.

**ITGB1**, a prominent HCCS15 gene, together with **ITGA3**, which is a prominent HCCS24 gene (Figure 3), generate an integrin heterodimer that links the cell surface to ECM structures such as focal adhesions [59] (Figure 12, *interaction 18*). Dynamic local binding and release of integrins from ECM proteins is required for cells to move relative to extracellular matrix. Interactions between ITGB1 and ADAM9 have already been noted (see section on ADAM9). Integrins bind many proteins on the cytoplasmic side of the membrane and bind matrix proteins on the extracellular side [60]. Interactions on either side affect interactions on the other side (inside-out and outside-in signaling). Integrins also serve as mechanical connector between ECM and cytoskeleton. The mechanical and signaling pattern on the cytoplasmic side can switch between different binding partner arrangements that can link directly or indirectly to the integrins [60].

**CD151**, a HCCS24 gene (Figure 3), encodes a tetraspanin transmembrane protein that binds integrins, such as ITGA3-ITGB1 (Figure 12, *interaction 19*), and facilitated cell migration [61]. CD151 promotes tumor cell motility, invasion and metastasis of several clinical cancers. The enhanced mobility of hepatic carcinoma cells was dependent on CD151-ITB1 interaction [62]. CD151 binds directly to ITGB1 and ITGA3, and the complex binds indirectly to THSB1 (thrombospondin), a HCCS24 gene product (Figure 3) [62] (see section on THSB1 below). An extracellular region of CD151 binds a membrane-proximal ectodomain of ITGA3. The structural factors involved in this action were recently analyzed with the view of potential development of therapeutic strategies whereby cell migration could be inhibited while leaving stable cell attachments intact [61]. Expression of CD151 was found to correlate with expression of MMP9, and both correlated with poor prognosis in hepatocellular carcinoma [63].

**LOXL2**, a HCCS24 gene (Figure 3), encodes a secreted copper-dependent lysyl oxidase that crosslinks collagen and elastin [64]. LOXL2 promotes tumor cell invasion and is associated with metastasis and reduced survival of patients with various cancers. Its expression in tumor cells or tissues is associated with MMP9 and with another matrix metalloproteinase, TIMP1. Crosslinking of collagen by LOXL2 may stimulate matrix metalloproteinases such as MMP9 and TIMP1 to implement ECM remodeling (Figure 9, *interaction 20*), thereby facilitating tumor cell migration [64]. LOXL2 is a direct transcription target of HIF1A [65], whereby it is up-regulated in response to hypoxia (*interaction 21*). If VHL (Von Hippel-Lindau gene) is defective, HIF1A and LOXL2 are expressed even in normoxia [65].

**AMOTL2** (angiomotin-like 2), encoded by a HCCS24 gene (Figure 3), down-regulates YAP1 [66] (*interaction 22*), which is a transcription co-activator of AXL [67] (*interaction 23*). AMOTL2 may suppress an epithelial-mesenchymal transition promoted by YAP1.

***EGFR*** (epidermal growth factor receptor) was one of the most prominent HCCS24 genes (Figure 3); it correlated unusually strongly with the expressions of ADAM9, AXL, and ITGB1 (expression profile correlations 0.76–0.78), in accord with findings that EGFR interacts with a variety of integrins [59] (Figure 12, *interaction 24*). EGFR and integrin each have the ability to affect the downstream actions of the other, although the pathway details have not been clarified. Beta1-integrins can promote EGFR-dependent cell proliferation stimuli and might be developed as a therapeutic target [68]. ADAM9 can cleave the precursor of EGFR ligand NRG1 on the surface of an adjacent cell to release NRG1 into the extracellular space, where it can bind and activate EGFR [40] (*interactions 25–27*).

***THBS1*** (thrombospondin) is a disulfate-linked glycoprotein involved in cell-to-cell and cell-to-matrix communications. It is associated with ECM and interacts with a variety of plasma membrane proteins, including some beta-1 integrins (Figure 12, *interactions 28* and *29*). It may modulate ECM-integrin association [69], either enhancing or weakening local cell-ECM attachments, thereby allowing cells to be mobile [70]. THBS1 binds CALR (calreticulin) in a Ca(2^+^) or Zn(2^+^) dependent manner, as a consequence of which adhesions between ECM and integrins are disrupted [71]–[74] (*interaction 30*).

The THBS1 promoter has 3 AP-1 sites, suggesting that the gene could be stimulated by a complex of **JUN** and a FOS-family protein, perhaps including **FOSL2** (*interaction 31*). Indeed, THBS1 transcription was activated by phosphorylation of JUN by MAPK8/JNK1 [75]. That could account for the highly correlated expression of THBS1 with JUN and FOSL2 (expression profile correlations 0.66 and 0.60, respectively; Figure 3). MAPK8/JNK1-induced JUN phosphorylation is counteracted by phosphatase **DUSP1** (*interactions 32* and *33*). JUN, FOSL2, and DUSP1 are in HCCS24 (Figure 3), and the latter two genes are also in HCCS15 (Figure 2).

THBS1 gene expression in the NCI-60 was highly correlated with dermatan sulfate epimerase (DSE) and chondroitin sulfotransferase CHST3 (Figure S1). This is of interest, because THBS1 can bind proteoglycans such as chondroitin, dermatan, and heparan sulfates [76], and these are constituents of extracellular matrix. These interactions will be discussed in a forthcoming report.

THBS1 is expressed in the developing central nervous system (CNS), promotes neurite outgrowth from cultured neurons, and is implicated in the migration of neural precursor cells. [77]. Notably, THBS1 was strongly expressed in all 6 CNS cell lines in the NCI-60 panel (Figure 4). Moreover, it is plausible that neurite extension would employ some of the same molecular interactions as cell migration.

**MYH9** is a high cross-correlation HCCS15 gene (Figure 2) that codes for the heavy chain of non-muscle myosin IIA. Mutations or allelic variations of MYH9 are associated with platelet pathology and defective podocyte function in renal glomeruli [78]–[79]. The association with kidney disorders fits with a high expression of MYH9 that we observed in the NCI-60 renal cancer lines (Figure 4). The MYH9 gene expression profile was also strongly correlated with the metastasis-related gene THBS1/thrombospondin (expression profile correlation 0.67; Figure 3).

In the polarized migrating cell, myosin II accumulates in the sides and rear, while F-actin tends to enrich in the front of the cell; the two engage in coordinating forward extension and rear retraction [80]–[81]. Myosin IIA may release adhesions at the rear part of the migrating cell by relaxing adhesion-stabilizing stress fibers [82]. An association between MYH9 and cell migration is also suggested by the effect of microRNA let-7f, which down-regulates MYH9 expression, as well as cell invasion and metastasis, [83]. (Let-7f expression however did not correlate significantly with the migration-associated HCCS15 genes in the NCI-60 cell lines.)

AXL has been reported to interact with non-muscle myosin heavy chain IIB (MHC-IIB) [84]. Since MYH9 codes for non-muscle myosin heavy chain IIA and its expression correlated exceptionally well with AXL (expression profile correlation 0.74; Figure 2), we conjecture that AXL and MYH9 in myosin IIA may associate with each other and function together to confer movement to migrating tumor cells, particularly in the process of rear retraction, for which myosin IIA is required in several cell types [11] (Figure 12, *interactions* 34 and 35).

### Gene expression related to migration, mesenchymal, and epithelial characteristics

The epithelial-to-mesenchymal transition (EMT) is characterized by increased expression of VIM (vimentin) and decreased expression of E-cadherin (CDH1) [85]. Thus VIM is commonly taken as a marker of mesenchymal and CDH1 of epithelial character. Accordingly, nearly all of the NCI-60 cell lines exhibit an inverse relationship between the expression of VIM and CDH1, indicating which lines are epithelial, mesenchymal, or indeterminate (Figure 6C). These distinctions can also be seen in the clustered image map (CIM) in Figure 4. The cell lines that strongly express the high cross-correlation genes associated with cell migration (HCCS24; Figure 3) are all in clusters D, E1, or E2 in the CIM (Figure 4). Most of these cell lines express VIM more strongly than CDH1 and therefore presumably have mesenchymal character. In cluster A, we see the cell lines that express the migration-related genes only weakly. Most of these lines express CDH1 more strongly than VIM and therefore presumably have epithelial character. Within this cluster, however, most of the melanoma lines express VIM more strongly than CDH1, despite their low expression of migration-related genes. It seems therefore that melanoma cells may often have mesenchymal properties while lacking the cell migration mechanisms commonly employed by other solid tumors.

Ovarian carcinoma cell line OVCAR4 was unusual in having high expression of both CDH1 and VCL (figure 4, arrow). (VCL expression correlated well with both mesechymal and cell migration related genes.) OVCAR4 therefore may have epithelial character and engage in cell-cell junction activity enhanced by VCL binding alpha and/or beta catenin, producing the bridge CDH1-catenin-VCL-actin [35]. OVCAR4 showed high expression also of AMOTL2 and COL4A1 (Figure 4, arrow), suggesting high potential for collagen production (Figure 12).

The gene expression profile of AXL, which is at the top of the list of migration-related gene (Figure 3), was remarkably similar to that of the mesenchymal marker genes VIM and SNAI2 (Slug), with some exceptions, most notably by melanoma lines that strongly expressed VIM and SNAI2, but not AXL (Figure 6D). AXL and VIM are up-regulated together in migrating cells at monolayer wound edges and in breast carcinomas [86]. VIM and SNAI2 were highly expressed together with AXL in breast cancer cell lines MDA_MB_231, HS578T, and BT_549, for example, and may confer mesenchymal-like character to these lines (Figure 6D).

Cluster B near the center of the CIM is made up largely by leukemia lines (Figure 4), which exhibit low expression of both VIM and CDH1 and very low expression of most of the migration-related genes.

The gene-gene expression correlations are shown in the CIM in Figure 5. The high cross-correlations of the HCCS24 genes (upper 24 genes in Figure 3) are evident in the almost uniformly red area enclosed in the blue box in Figure 5. Of interest here is that some genes, such as RAC1 and GAS6, that were not sufficiently cross-correlated to be included in HCCS24 nevertheless correlated significantly with many of those HCCS24 genes. Only FURIN and FAM38A failed to correlate well with the entire set. For FURIN, that is attributable to its functioning in adjacent stromal cells rather than the tumor cells (Figure 12).

### Comparison between NCI-60 cell migration-related genes and EMT-upregulated genes in NSCLC cell lines [87]

Cell migration requires the coordinated function of several processes, only a few of which were described above. For epithelial cells to migrate, they must first undergo an epithelial-mesenchymal transition (EMT) involving molecular changes that have recently been characterized by transcriptional and proteomic analysis by Thomson et al [87]. Of approximately 50 genes/proteins that they found to be up-regulated during EMT in genetically engineered NSCLC cells, FLNA, THBS1, ITGB1, and LOXL2 were in our HCCS24 high cross-correlation set (Figure 3); in addition, TPM1 was in the extended HCCS66 gene set (Figure S1). The limited number of genes co-existing in both gene sets is presumably due to the major differences in the systems and methods employed. It probably reflects the large number of different systems that participate in various aspects of cell migration.

**Figure 13:**
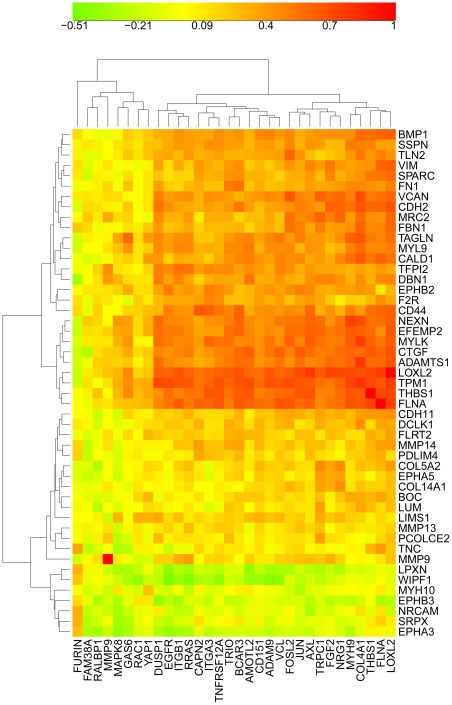
Relationship between cell migration-related genes in this report with EMT-upregulated genes reported by Thomson et al [87]: Clustered image map (CIM) of the cell migration-related genes (horizontal) listed in Figure 3 versus the EMT-upregulated genes (vertical) reported by Thomson et al (reddish color in their Figure 3). This CIM depicts the expression correlations between the respective genes in the NCI-60 cell line panel.

To show the relationship between the EMT-upregulated genes of Thomson et al and the cell migration-related genes listed in Figure 3, we prepared a clustered image map of the NCI-60 gene expression cross-correlations between those two sets of genes (Figure 13). We see that the EMT-upregulated genes divided into clusters, depending on how well they correlated with the NCI-60 gene migration-related gene set of Figure 3.

**Figure 14:**
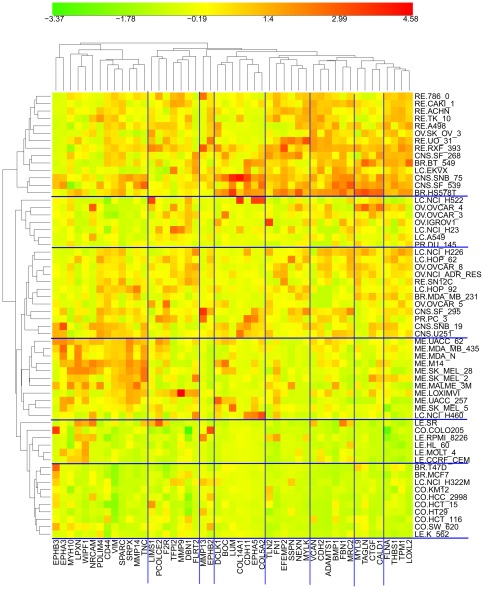
Relationship between cell migration-related genes in this report with EMT-upregulated genes reported by Thomson et al [87]: CIM of the expression of the EMT-upregulated genes in the NCI-60 cell line panel.

We also prepared a clustered image map of the expression of the EMT-upregulated genes in the NCI-60 cell lines (Figure 14). We see a marked dependence on cell type: epithelial (CO = colon, breast T47D and MCF7; LE = leukemia; ME = melanoma). The upper cluster contains mesenchymal-like cell lines, based on high VIM/CDH1 expression (the epithelial-like cell lines had high CDH1/VIM expression ratios). (CDH1 was included to show epithelial character.)

These findings illustrate the complexity of the EMT and cell migration-related regulation networks that remain to be elucidated, and suggest that those networks relate to each other in definable ways.

## Discussion

Tissue invasion and metastasis require molecular perturbations that give tumor cells the mobility to migrate inappropriately. This is one of at least 10 sequential changes that must occur for most solid tumors to become fully malignant [10]. Migration of malignant cells seems to differ from most normal cells in a more rapid turnover of focal adhesions and more effective proteolysis of extracellular matrix [30].

Cell migration is controlled via an extensive and highly connected network of interactions between integrins and cytoskeleton, as well as between integrins and the cell exterior. Thus the migration of adherent cells involves a complex interplay between structural changes and signaling. The very large number of interacting molecules in these processes constitute an ‘adhesome’ that presumably provides both robustness and flexibility [60]. The sheer size and complexity of this network however makes efforts to understand it a daunting task. We have made the problem more tractable by putting the spotlight on a limited area of the full network by focusing on those molecules whose genes are coexpressed in a particular set of cell lines. The consequent molecular interaction maps (Figures 11 and 12) therefore describe a defined part of the full migration-related network: they describe cell migration processes occurring in certain cells under certain circumstances (although precisely when those circumstances apply remains to be determined).

The current study identified cell migration-related genes that were correlated both in expression profiles and in molecular interactions and that comprised networks that we portrayed as molecular interaction maps (Figures 11 and 12). We explored the premise that gene expression correlations at the mRNA level can reflect gene function relationships within networks of molecular interactions. From a set of genes that have correlated gene expression profiles in the NCI-60 panel of human tumor cell lines, together with published information on their (or their product's) molecular interactions, we generated network models of how those interactions may lead to coherent function. It seems remarkable how expression-correlated genes came together in interaction networks of coherent function (Figures 11 and 12).

We began with 15 highly cross-correlated genes (HCCS15) (Figure 2), which were a subset of a cluster of 83 expression-correlated genes derived by Zeeberg et al [9] that were enriched in Gene Ontology (GO) categories loosely related to cell motility (Figure 1). We then used the HCCS15 genes as seeds to harvest other genes whose expression profiles were highly correlated with them. This procedure yielded a set of 66 cross-correlated (HCCS66) (Figure S1), which we then surveyed for molecular interactions and function reported or reviewed in current literature. Most of the HCCS66 genes had reported interactions or functions that related to several different aspects of cell migration mechanisms and that could be depicted as molecular interaction maps (MIMs) [12], [13] (http://discover.nci.nih.gov/mim/). Here we developed network models for the role of RRAS and calcium (Figure 11), and for interactions between extracellular matrix and the cell surface (Figure 12). These particular migration-related functions implicated 24 genes (HCCS24), which are a subset of the 66 genes in HCCS66. (HCCS66 includes genes involved in other migration-related functions that may be the subject of a subsequent report). Relevant information for each gene is summarized in sections of text in the Results. We aimed to relate the expression correlations with the molecular interactions and functions in the greatest possible detail in accord with current evidence and hypotheses. In addition, we noted function relationships that may not have been previously noted, including evident, unexpected relationships for further investigation.

The first question we addressed was whether a correlation of gene expression could predict an unexpected function relationship. We noted an unusually high expression correlation between the HCCS15 genes RRAS and ITGB1 (beta1-integrin) (expression profile 0.78) (Figures 2 and 6A). We initially did not know how those genes could be functionally related. However, a literature search for reports dealing with both genes retrieved a recent paper that connected them. in regulation of integrin-actin linkage by calcium efflux from endoplasmic reticulum [20]. The authors proposed a model of how RRAS and ITGB1 function to regulate the linkage between extracellular matrix (ECM) and the cell surface. We depicted the proposed model as a MIM in Figure 8. A key step in the model was activation of calpain by calcium, but it has not been determined which of the calpains may be involved. From the expression correlations of 11 calpain isoforms with the HCCS15 genes, we inferred that the relevant isoform was probably calpain-2 (CAPN2) (Figure 10).

We then built upon the model by adding relevant interactions of genes from HCCS66 (see *Methods*), depicted as a MIM in Figure 11. The network depicted in Figure 11 has 15 genes/products, of which 9 (shown in red) were highly expression-correlated with other migration-related genes and were included in HCCS24 (a subset of HCCS66 comprised of the genes relevant to the current coverage). This network, which has well-defined functions in cell migration, thus is composed largely of expression-correlated genes. The network is centered on the actions of RRAS and calcium on the linkage between integrins and the actin cytoskeleton. The integrated functions of the genes in this network (Figure 11) can be summarized as follows. (The highly expression-correlated genes/products are in bold in the text below; interaction details and references are given under *Results* in the summaries for the individual genes.)

**RRAS**, which can be activated by **BCAR3**, has several actions in this network. First, it stimulates release of calcium from endoplasmic reticulum (ER); if ER calcium is depleted, STIM1, which senses calcium concentration in the ER, stimulates TRPC1 [31]. **TRPC1** helps to open channels that allow calcium entry into the cell. The elevated cytosolic calcium activates calpain **CAPN2** to abrogate the linkage between integrin and cytoskeleton. Second, **RRAS** is recruited by filamin **FLNA** to integrin **ITGB1** and the actin cytoskeleton to enhance the binding of integrin to extracellular matrix (ECM). Third, **RRAS** may assist in temporary endocytosis of integrins, a process that could function cooperatively in the adhesion/de-adhesion cycle. Finally, **RRAS** acts via the RAC1 pathway to stimulate the production of cell surface structures that interact with ECM and promote cell mobility.

We further extended the picture by adding another HCCS24-based network of the regulation of ECM-cell surface attachments, depicted as a MIM in Figure 12. This network involves 26 genes/products, 17 of which had high expression cross-correlations and are included in HCCS24. The focus of this network is degradation of ECM proteins by matrix metalloproteinases (MMPs), whereby attachments between cell surface and ECM are disrupted. The MIM shows how this ECM degradation process may be coordinated though the actions **AXL**, **ADAM9**, and **TNFRSF12A**/FN14. AXL and ADAM9 up-regulate matrix metalloproteinases via the ERK pathway, while AXL and TNFRSF12A/FN14 do so via the NFkB pathway. MMPs then degrade ECM, so as to weaken the attachment of cells to substratum, thereby freeing cells to migrate.

ECM degradation in the tumor environment, however, may be due largely to MMPs secreted by neighboring fibroblasts rather than by the tumor cells themselves [88]. Figure 12 outlines some pathways whereby this may occur. The activation of MMP transcription by NFkB may be promoted when **AXL** on the cell surface binds its extracellular ligand, GAS6. Alternatively, this activation may be promoted when FN14 (a product of the **TNFRSF12A** gene) on the cell surface binds TWEAK on the surface of an adjoining cell. This intercellular promotion activity could occur in either direction between tumor and stromal cell. Thus the inter-cellular interactions of AXL, FN14, and ADAM9 may be important in regulation of coupling between tumor and stromal cells.

We think it remarkable that the coherent network models that are portrayed as molecular interaction maps in Figures 11 and 12 consist largely of genes (or gene products) whose expression profiles are highly cross-correlated over diverse cell lines (Figures 3–5). The network model in Figure 12 depicts controls on 3 events required for cells to be able to migrate: degradation of extracellular matrix, production of focal complexes at the leading edge of the cell, and retraction of the rear part of the cell. Figure 11 depicts controls on Ca (2^+^), which may govern those events in a spacio-temporally localized manner in the cell [23], [89].

The highly cross-correlated genes we have discussed and mapped are expression correlated more highly with VIM (vimentin) than with CDH1 (E-cadherin) (Figure 5), and therefore tend to convey mesenchymal rather than epithelial character. That inference however applies only to those cell lines (the majority) that show an inverse expression: VIM high, CDH1 low (Figure 5); it does not apply to the leukemia lines nor to at least 2 of the melanoma lines (Figure 6C), for which no clear epithelial/mesenchymal assignment can be made based on CDH1/VIM ratio. This distinctive gene expression character of the leukemia lines is also evident in the clustered image map Figure 4. The close alignment of the high cross-correlation (HCCS24) genes with mesenchymal character is consistent with the functional interactions of these genes with extracellular matrix and stroma (Figures 11 and 12). In particular, the genes ADAM9, AXL, and TNFRSF12A may connect directly between mesenchymal tumor cells and stromal cells (Figure 12).

In conclusion, our findings on genes related to cell migration suggest how expression correlations can yield clues to molecular network functions. The results confirm many expected function relationships and, perhaps more importantly, suggest unexpected relationships for experimental investigation. Although mRNA assays may be imperfect indicators of expression at the protein level, we found that mRNA expression profiles by themselves did give substantial information about the role of corresponding proteins in interaction networks related to cell migration. More definitive results may be obtained with new technology, such as large-scale sequencing of ribosome-mRNA footprints [90], [91]. Although we found that genes having correlated expression profiles across a variety of human tumor cell lines tended to function together in the same molecular interaction network, the mechanism of the co-regulation remains to be elucidated.

## Supporting Information

Figure S1 **Extended high cross-correlation gene set HCCS66.** These 66 genes were selected on the basis of correlated expression with the HCCS15 genes of Figure 2 (see *Methods*). The gene names highlighted in yellow are the genes represented in the molecular interaction maps (Figures 11 and 12). (MIM symbol definitions are summarized in Figure 9.)
(TIFF)
