# Supplementary figures and images for: Gene Expression Profiles of the NCI-60 Human Tumor Cell Lines Define Molecular Interaction Networks Governing Cell Migration Processes
Source: PLoS One. 2012 May 3;7(5):e35716. doi: 10.1371/journal.pone.0035716 (PMC3343048; doi:10.1371/journal.pone.0035716)

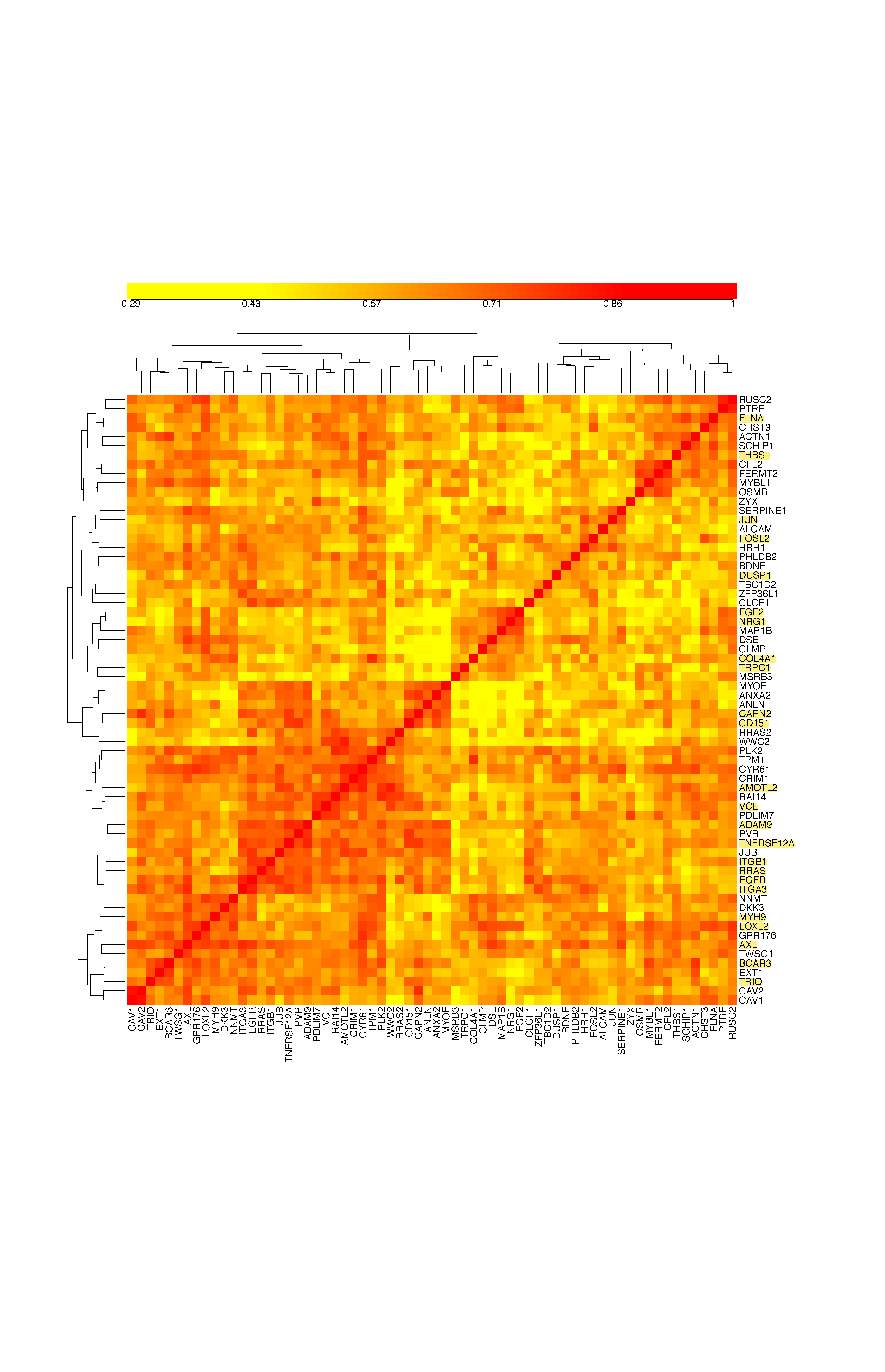

Supplement: Figure S1 — Extended high cross-correlation gene set HCCS66. These 66 genes were selected on the basis of correlated expression with the HCCS15 genes of Figure 2 (see Methods ). The gene names highlighted in yellow are the genes represented in the molecular interaction maps (Figures 11 and 12). (MIM symbol definitions are summarized in Figure 9.) (TIFF) [file pone.0035716.s001.tiff]
